# The Lithuanian Stroke Database: selection of national stroke care performance measures

**DOI:** 10.3389/fneur.2025.1550539

**Published:** 2025-05-23

**Authors:** Austėja Dapkutė, Justas Trinkūnas, Daiva Rastenytė, Vaidas Matijošaitis, Saulius Taroza, Dalius Jatužis, Sandra Baužaitė-Babušienė, Aleksandras Vilionskis, Andrius Klimašauskas, Julius Juodakis, Julius Jaramavičius, Rytis Masiliūnas

**Affiliations:** ^1^Clinic of Neurology and Neurosurgery, Institute of Clinical Medicine, Faculty of Medicine, Vilnius University, Vilnius, Lithuania; ^2^Department of Information Systems, Faculty of Fundamental Sciences, Vilnius Gediminas Technical University, Vilnius, Lithuania; ^3^Centre for Digital Medicine, Translational Health Research Institute, Faculty of Medicine, Vilnius University, Vilnius, Lithuania; ^4^Department of Neurology, Medical Academy, Lithuanian University of Health Sciences, Kaunas, Lithuania; ^5^Klaipėda University Hospital, Klaipėda, Lithuania; ^6^Department of Neurology, Republican Panevėžys Hospital, Panevėžys, Lithuania; ^7^Clinic of Anaesthesiology and Intensive Care, Faculty of Medicine, Institute of Clinical Medicine, Vilnius University, Vilnius, Lithuania; ^8^Clinic of Emergency Medicine, Faculty of Medicine, Institute of Clinical Medicine, Vilnius University, Vilnius, Lithuania

**Keywords:** stroke, quality improvement, stroke database, performance measures, stroke key performance indicators

## Abstract

**Introduction:**

The Lithuanian Stroke Database (StrokeLT) aims to automate data collection and key performance indicator (KPI) monitoring across all stroke-ready hospitals, addressing the limitations of manual processes and facilitating evidence-based improvements in stroke care nationwide. This publication outlines the selection process and target values of the KPIs designed to standardise and enhance stroke care quality in Lithuania.

**Study population:**

The database will include all adult patients diagnosed with stroke or transient ischemic attack (TIA), admitted to Lithuanian stroke-ready hospitals, encompassing approximately 9,582 annual stroke and 1,899 TIA admissions based on 2023 data. The database will ensure comprehensive national coverage by integrating data from stroke centres via a centralised electronic health record system.

**Main variables:**

A total of 53 KPIs were selected through a multi-stage Delphi process involving national experts and guided by international standards. These KPIs include 44 process metrics, such as timeliness metrics, early rehabilitation, and availability of secondary prevention, as well as 8 outcome metrics, including functional recovery, completion of a patient feedback survey and mortality. This framework enables comprehensive monitoring across all stages of patient care, as well as incorporating valuable patient feedback.

**Conclusion:**

The Lithuanian Stroke Database establishes a standardised automated framework for monitoring stroke care using 53 KPIs, selected through a multi-stage Delphi process involving all relevant stakeholders.

## Introduction

Stroke is one of the leading causes of death and disability worldwide (1) with Lithuania reporting one of the highest stroke incidences globally, amounting to 223.4 strokes per 100,000 inhabitants (2). In addition, Lithuania has been forecast to experience the largest increase in the age-adjusted stroke incidence and prevalence rates out of all European Union countries (3).

Effective stroke care relies heavily on timely interventions, adherence to guidelines, and multidisciplinary coordination – all of which can be monitored using key performance indicators (KPIs). Previous research has demonstrated that participation in quality improvement programs and registries leads to improved care processes and performance metrics (4, 5). In addition, it helps translate research evidence into clinical practice (6) and provides faster gains in patient-centred outcomes (7). Moreover, adherence to multiple KPIs has been proven to have an additive effect (6).

The Lithuanian comprehensive national policy for acute stroke care, implemented in 2014, has been shown to significantly improve a wide range of immediate and long-term stroke care quality measures (8). However, data collection for quarterly KPI analysis depends on manual labour by overqualified personnel, is time-consuming, and has a certain time lag. In addition, manual data collection limits the number of KPIs that could be monitored. Finally, manually submitted data is less reliable, potentially inducing bias in the performance assessment. Therefore, the stroke quality paradigm of the future states that reliable data should be retrieved from electronic health records (EHRs) automatically and integrated into reports used by frontline staff to monitor and address the needs of their patients without requiring excess documentation from clinical staff (9).

In April 2024, the Lithuanian State Enterprise Centre of Registers (SECR) (10) initiated the Data Exchange and Monitoring Platform for Medical Clusters – a national quality improvement project dedicated to developing regulated and standardised medical data monitoring across Lithuania. One part of this project was the establishment of the Lithuanian Stroke Database (StrokeLT), aimed at improving the quality and efficiency of stroke care across the country. The project is designed to generate and monitor unified indicators of healthcare service quality, essential for measuring active treatment outcomes, optimising patient care, and reducing time from symptom onset to treatment. The overarching goal of the stroke database is to move toward evidence-based healthcare management, ensuring that all relevant healthcare sectors – from emergency medical services to rehabilitation – are well-coordinated and equipped to provide high-quality care for patients with acute stroke. The database will enable automatic collection of real-time data, allowing for ongoing nationwide monitoring and evaluation of stroke services. This will lead to identifying areas for improvement and support informed decision-making for policymakers and clinicians alike.

This publication describes the selection process and target values of the KPIs chosen for the Lithuanian Stroke Database, designed to standardise and improve stroke care quality across the country.

## Setting

The Lithuanian healthcare system is funded through the compulsory National Health Insurance Fund, ensuring free access to state-funded health services, which, among others, encompass primary healthcare, emergency medical services (EMS), primary and secondary prevention programs, inpatient care, specialised outpatient care, prescription medications, and rehabilitation services (11). Stroke care in Lithuania is provided through a network of stroke-ready hospitals, established in 2014 as part of a national policy to improve stroke patient care in the country (8). There are six Comprehensive Stroke Centres (CSCs) and five Primary Stroke Centres (PSCs) strategically located to ensure patients can be admitted within the optimal therapeutic window for acute stroke interventions. All suspected stroke patients are required to be transferred to the nearest stroke centre by EMS, ensuring timely access to care.

In most cases, hospital information systems (HISs) differ between stroke-ready hospitals, as they could be acquired from different vendors or developed in-house. Nevertheless, all healthcare institutions are by law required to upload essential medical documents, such as discharge summaries, referrals, outpatient visits, diagnostic test reports, and electronic prescriptions, to the centralised Electronic Health Services and Cooperation Infrastructure Information System (Lith. *Elektroninės sveikatos paslaugų ir bendradarbiavimo infrastruktūros informacinė sistema*, ESPBI IS) controlled by the Ministry of Health of the Republic of Lithuania (11, 12). This makes patient EHRs available to all Lithuanian healthcare providers through information system integrations, as well as to patients through the Lithuanian eHealth patient portal free of charge (13). However, the clinical information in the centrally stored EHRs is mainly unstructured, i.e., in free text. While this is sufficient for patient information during clinical care, it is unsuitable for KPI calculation and monitoring. Some structured clinical parameters are stored in the HISs, but the format, standards and scope of such information differ between the hospitals.

SECR (10) is a public institution hosting and administrating various state registers, such as the Population Register, the Register of Legal Entities, and many others. It also creates, develops, and administers information systems of national importance, such as the abovementioned ESPBI IS, and provides register data to government institutions and private entities.

**Figure 1:**
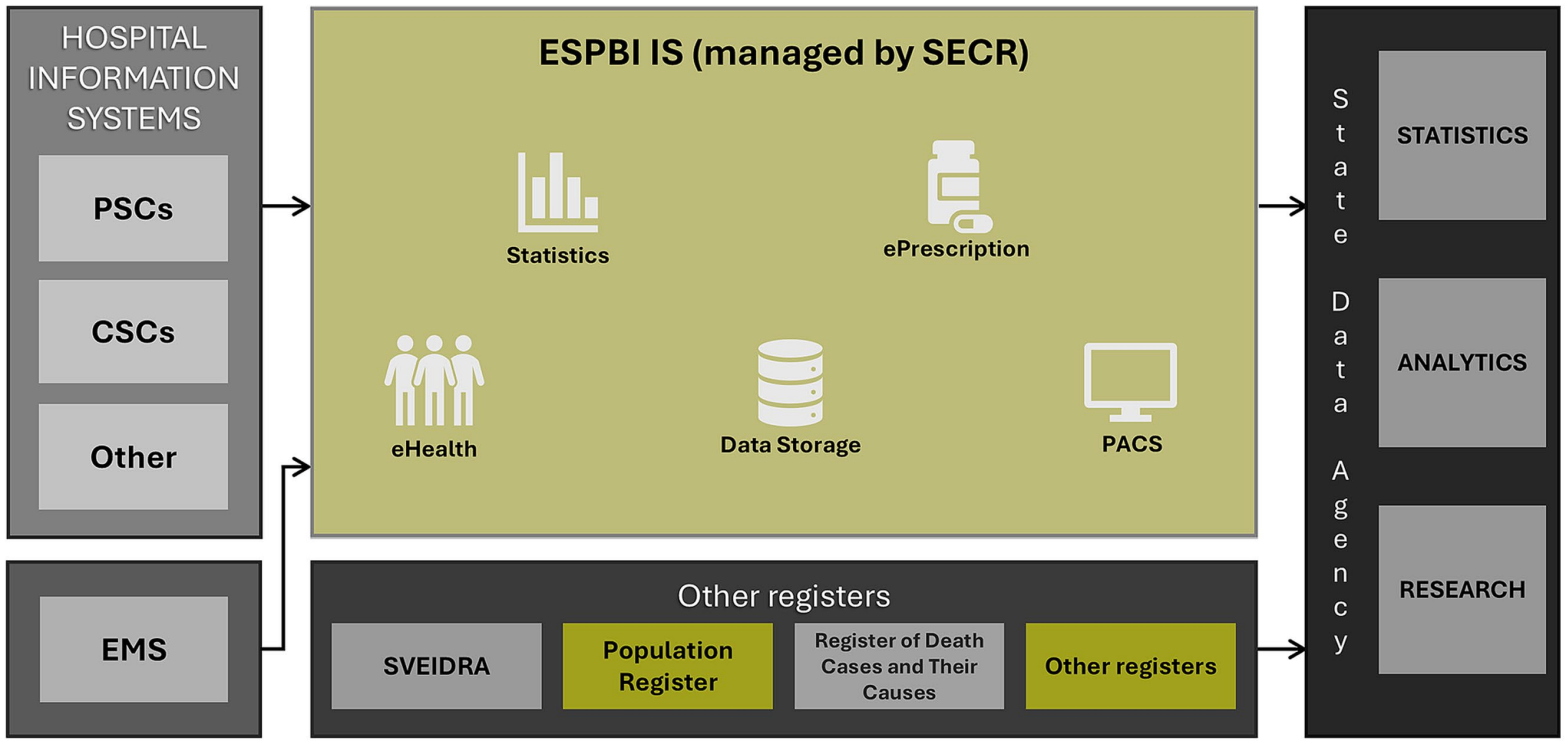
Lithuanian Stroke Database data flow. PSC, primary stroke centre; CSC, comprehensive stroke centre; SECR, State Enterprise Centre of Registers; PACS, Picture Archiving and Communication System; ESPBI IS, State Electronic Health Services and Cooperation Infrastructure Information System; SVEIDRA, Compulsory Health Insurance Fund information system. Entities in yellow represent registers managed by SECR.

Initiated by SECR, the national quality improvement project, of which the Lithuanian Stroke Database is a part, makes use of the available data from the state registers, ESPBI IS, and directly from participating stroke centres, EMS, as well as other healthcare institutions (Figure 1). The project will fund the identification and programming of new modules for each HIS so that the data relevant to selected stroke care KPIs can be collected from the stroke-ready hospitals in an automated way to make the dataset standardised, structured, and shareable. The collected data will reflect the entire continuum of stroke care, from acute treatment through rehabilitation and stroke outcomes.

## Study population

According to official statistics, the Lithuanian population totalled 2,885,891 inhabitants at the beginning of 2024, with 1,365,905 males (median age of 40 years, life expectancy 72.9 years) and 1,519,986 females (median age of 47 years, life expectancy 81.7 years) (14). The Lithuanian Stroke Database will prospectively include all adult patients (aged 18 years or older) admitted with a diagnosis of stroke or transient ischemic attack (TIA) to any Lithuanian hospital, including the 11 Lithuanian PCSs and CSCs. Stroke and TIA patients are identified by diagnoses from discharge summaries and outpatient records based on the International Classification of Diseases, Tenth Edition, Australian Modification (ICD-10-AM) codes, with stroke cases classified under I60-I61 and I63-I64 and TIA cases under G45 (except G45.4, which codes for transient global amnesia). By law, stroke must be diagnosed in a PSC or CSC, ensuring comprehensive national coverage of all emergency department and hospital admissions.

The precise number of patients to be included annually cannot be determined in advance. However, retrospective Lithuanian insurance fund data suggest that there were 1,899 TIA admissions and 9,582 stroke admissions in 2023, 8,190 of which were ischemic strokes (15).

## Quality indicators

**Figure 2:**
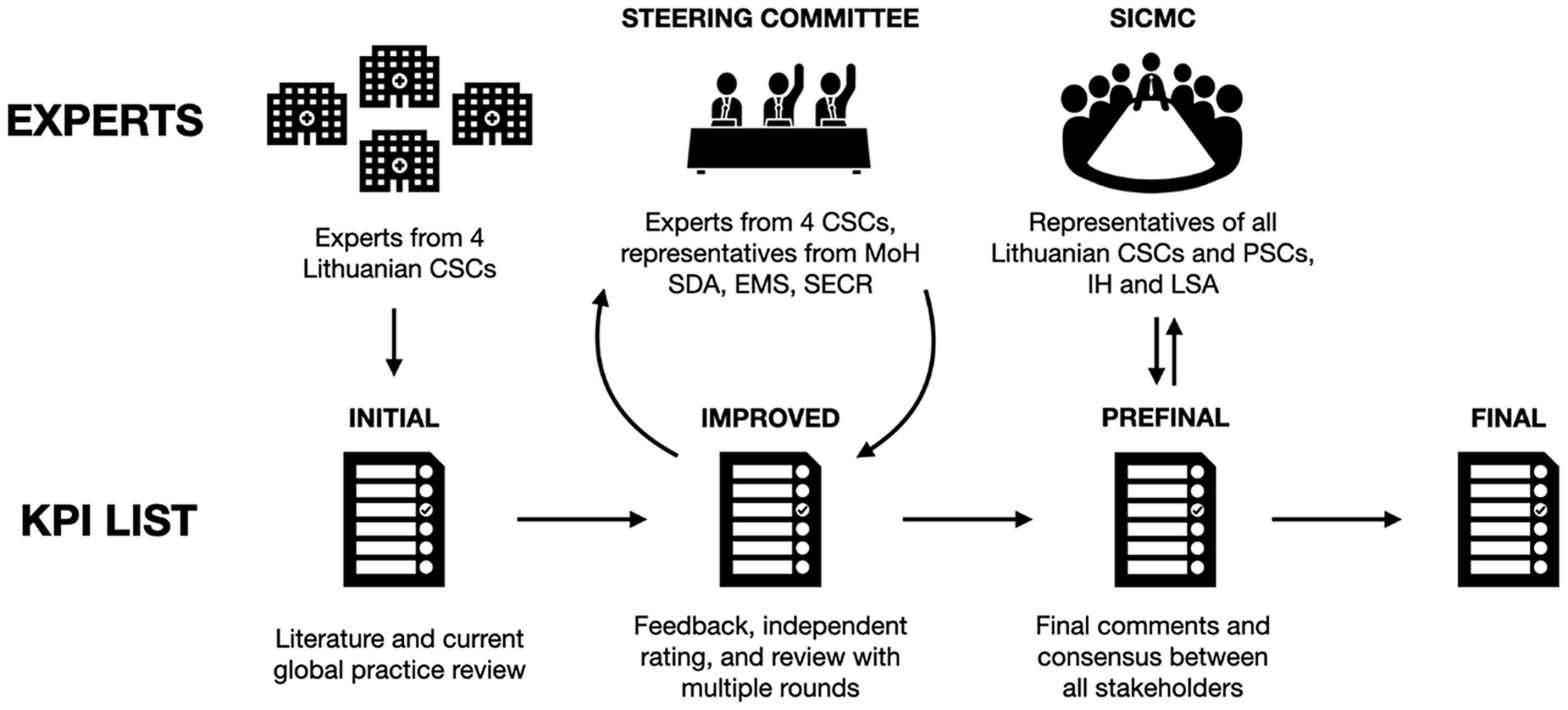
Outline of the Delphi process, adapted for selecting key performance indicators for the Lithuanian Stroke Database. CSC, Comprehensive Stroke Centre; MoH, Ministry of Health; SDA, State Data Agency; EMS, Emergency Medical Services; SECR, State Enterprise Centre of Registers; SICMC, Stroke Integrated Care Management Committee; PSC, Primary Stroke Centre; IH, Institute of Hygiene; LSA, Lithuanian Stroke Association.

A total of 53 KPIs were rigorously selected for the Lithuanian Stroke Database, following a Delphi process, adapted for the setting (16) (Figure 2).

The initial literature review and selection of the primary set of KPIs were performed by experts from four CSCs representing the four major types of HISs used by the Lithuanian hospitals, namely Vilnius University Hospital Santaros Klinikos, Hospital of Lithuanian University of Health Sciences Kauno Klinikos, Klaipėda University Hospital, and Panevėžys Republican Hospital. The initial set consisted of KPIs defined by international guidelines and registries, including those of the European Stroke Organisation (ESO) (17), the American Heart Association (AHA) (7), the National Institute for Health and Care Excellence (NICE) (18), SAP-E (Stroke Action Plan for Europe) (19), and the Danish Stroke Register (20). These registries were suggested by CSCs’ experts based on their widespread use and recognition in the field of stroke care. The Danish Stroke Register was included specifically as an example of automatic stroke KPI monitoring that has already been implemented in settings geographically and demographically similar to Lithuania, making it a relevant reference for the development of our national database. Indicators were prioritised based on clinical relevance, feasibility of automated extraction, and data reliability within the existing HIS infrastructure, with future expansion planned as data integration improves.

The set was independently evaluated by the Steering Committee, which also included representatives from the Lithuanian Ministry of Health, State Data Agency (SDA), Institute of Hygiene, and SECR. A total of 13 stakeholders contributed, including neurologists, stroke unit leads, health informatics specialists, and policymakers, with seniority ranging from experienced clinicians to national-level decision-makers (see Supplementary Table 1), although no patients or patient representatives were involved. The process was conducted through a combination of face-to-face and online meetings, with mandatory participation in each round, with a 100% response rate throughout the process. Evaluations were analysed, feedback was provided on the responses, and experts revised their assessment. The process was repeated for eight rounds until a consensus was reached, with all stakeholders voting unanimously.

The chosen set of KPIs was then presented to the Stroke Integrated Care Management Committee (SICMC; for the list of representatives, see Supplementary Table 2), where it was reviewed and commented on by the delegates of the remaining Lithuanian CSCs and PCS, EMS, and members of the Lithuanian Stroke Association. Six individuals participated in both the Delphi process and the SICMC. Following the presentation, two additional KPIs were added, and three underwent minor adjustments. These changes did not alter the overall focus of the KPI set. After incorporating the revisions, the final list of StrokeLT KPIs was compiled, and targets were set where applicable (Table 1).

**Table 1:** Indicators and standards of the Lithuanian Stroke Database (StrokeLT).

Indicator area	Indicator	Type	Standard
Diagnosis	Number of hospital-treated acute stroke patients by stroke type	Process	N.S.
	Number of TIA patients	Process	N.S.
	Proportion of EMS-suspected stroke/TIA patients whose diagnosis was confirmed at PSC or CSC	Process	N.S.
Prenotification	Proportion of cases when EMS prenotified PSC or CSC about a suspected stroke arrival	Process	100%
Fast admission	Median time from stroke symptom onset to arrival at PSC or CSC	Process	<120 min.
	Median time from the first contact with EMS to arrival at PSC or CSC	Process	<60 min.
	Proportion of suspected stroke cases transferred to PSC or CSC within 60 min of an emergency call	Process	100%
	Proportion of acute stroke patients in Lithuania diagnosed at PSC or CSC	Process	>95%
Hospital admission	Proportion of acute stroke patients treated at PSC or CSC in Lithuania	Process	>90%
	Median length of hospital stay for acute stroke patients	Process	N.S.
Imaging	Proportion of stroke patients receiving CT or MR scan at PSC or CSC	Process	100%
	Median time from acute stroke patient arrival to PSC or CSC to imaging (CT/MR)	Process	<30 min.
	Proportion of acute stroke patients receiving CT within 30 min of arrival to PSC or CSC	Process	100%
	Proportion of acute ischemic stroke patients receiving CT angiography	Process	N.S.
Intravenous thrombolysis	Number of acute ischemic stroke patients treated with IVT	Process	N.S.
	Proportion of acute ischemic stroke patients treated with IVT overall in Lithuania	Process	>15%
	Median time from stroke symptom onset to IVT	Process	<120 min.
	Median time from acute ischemic stroke patient arrival to PSC or CSC to IVT administration	Process	<40 min.
	Proportion of patients receiving IVT within 60 min of arrival to PSC or CSC	Process	95%
Endovascular treatment	Number of acute ischemic stroke patients treated with EVT	Process	N.S.
	Proportion of acute ischemic stroke patients treated with EVT	Process	>5
	Median time from stroke symptom onset to initiation of EVT	Process	<200 min.
	Median time from acute ischemic stroke patient arrival to PSC or CSC to initiation of EVT	Process	<90 min.
	Proportion of acute ischemic stroke patients treated with EVT within 120 min of arrival	Process	>90%
	Median time from acute ischemic stroke patient arrival to PSC to departure to CSC for EVT	Process	<60 min.
	Proportion of acute ischemic stroke patients departing from PSC to CSC for EVT within 60 min of arrival at PSC	Process	>95%
	Proportion of acute ischemic stroke patients treated with EVT achieving reperfusion mTICI ≥2C	Process	>80%
Reperfusion therapy	Number of acute ischemic stroke patients treated with both IVT and EVT	Process	N.S.
	Proportion of acute ischemic stroke patients treated with both IVT and EVT	Process	N.S.
	Proportion of acute ischemic stroke patients treated with IVT and/or EVT	Process	N.S.
Neurological assessment	Proportion of stroke patients with baseline NIHSS assessment completed	Process	>95%
	Proportion of stroke patients with NIHSS completed at 24 h ± 6 h after the initial assessment	Process	>95%
	Proportion of stroke patients with NIHSS completed at 7 days ± 24 h after the initial assessment	Process	>95%
	Proportion of stroke patients with NIHSS completed at discharge form hospital	Process	>95%
Functional assessment	Proportion of stroke patients with baseline mRS assessment completed	Process	>95%
	Proportion of stroke patients with mRS completed at 7 days ± 24 h after the initial assessment	Process	>95%
	Proportion of stroke patients with mRS completed at discharge from hospital	Process	>95%
Secondary prevention	Proportion of acute ischemic stroke patients receiving antithrombotic therapy within 48 h of hospital admission	Process	>95%
	Proportion of acute ischemic stroke or TIA patients without AF receiving antithrombotic therapy at discharge from hospital	Process	>90%
	Proportion of acute ischemic stroke or TIA patients with AF receiving anticoagulants at discharge from hospital	Process	>90%
	Proportion of acute ischemic stroke patients with AF on anticoagulants 1 year after the initial prescription	Process	>95%
Timely rehabilitation	Proportion of stroke patients receiving rehabilitation within 72 h of hospital admission	Process	>90%
	Median time from stroke patient discharge from hospital to start of rehabilitation	Process	0 days
	Proportion of stroke patients referred to rehabilitation	Process	N.S.
Screening for dysphagia	Proportion of stroke patients receiving swallowing function assessment within 4 h of hospital admission	Process	>90%
Mortality	Number of stroke patients who died while in hospital	Outcome	N.S.
	Proportion of acute ischemic stroke patients who died while in hospital	Outcome	<10%
	Proportion of patients with intracerebral haemorrhage who died while in hospital	Outcome	<30%
	Proportion of patients with subarachnoid haemorrhage who died while in hospital	Outcome	<30%
	Proportion of acute ischemic stroke patients who died within 30 days of the index event	Outcome	N.S.
	Proportion of patients with intracerebral haemorrhage who died within 30 days of the index event	Outcome	N.S.
	Proportion of patients with subarachnoid haemorrhage who died within 30 days of the index event	Outcome	N.S.
Patient involvement	Proportion of patient satisfaction surveys completed	Outcome	>90%

N.S., Not Set; TIA, Transient Ischemic Attack; EMS, Emergency Medical Services; PSC, Primary Stroke Centre; CSC, Comprehensive Stroke Centre; CT, Computed Tomography; MR, Magnetic Resonance; IVT, Intravenous Thrombolysis; EVT, Endovascular Therapy; mTICI, modified Thrombolysis in Cerebral Infarction scale; NIHSS, National Institutes of Health Stroke Scale; mRS, modified Rankin Scale; AF, Atrial Fibrillation.

Performance indicators used in healthcare usually fall into one of the four categories: structure, process, efficiency (i.e., cost-effectiveness), and outcome (21). Most selected StrokeLT KPIs focus on clinical processes – such as door-to-needle times, adherence to early rehabilitation within 24 h, and secondary prevention treatments. Additionally, some also assess stroke outcomes, including functional recovery at discharge, mortality within 30 days of hospital admission, and long-term disability reduction, measured as care results. To complement clinical and outcome KPIs, patient-centred care quality will be assessed through a patient satisfaction survey conducted at discharge. This comprehensive set of process- and outcome-oriented metrics ensures that stroke care in Lithuania is aligned with internationally recognised standards, promotes continuous quality improvement, and integrates patient feedback. In addition to the main KPIs, StrokeLT will also enable to perform cross-sectional analysis regarding the variables of interest. The key variables in the Lithuanian Stroke Database are presented in Table 2.

**Table 2:** Key variables in the Lithuanian Stroke Database.

Variable	Values
Demographics	
National Identification Number	Numeric
Date of birth	Date
Date of death	Date
Sex	Male/Female
Health-related information	
Previously prescribed medications	ATC subgroup
Earlier diagnoses	ICD-10 codes
Mechanical valve implanted	Yes/No
Atrial fibrillation recorded in history	Yes/No
Previous brain aneurysm treatment	Endovascular/Surgery/None/N.A.
mRS before stroke	0–5
Stroke-related information	
Stroke type	ICD-10 code
TIA type	ICD-10 code
Mode of arrival	EMS/Private transport/Self-presented
Remote consultation before the arrival	Yes/No
Diagnosis establishment setting	Inpatient/ER/Outpatient
Type of medical care establishment	CSC/PSC/Other
Transfer by EMS	Yes/No
Time of the first contact with EMS	Date, time
Transfer from PSC to CSC	Yes/No
Last known well	Date, time
Time of arrival at PSC	Date, time/N.A.
Time of discharge from PSC	Date, time/N.A.
Time of arrival at CSC	Date, time/N.A.
Inpatient admission to CSC	Yes/No
Inpatient admission to PSC	Yes/No
Time of inpatient admission	Time
Referral to a regional hospital for inpatient admission	Yes/No
Reperfusion therapy	IVT/EVT/Both/None
Time of IVT initiation	Date, time
IVT medication	Alteplase/Tenecteplase/Other/N.A.
Time of groin puncture (MTE)	Date, time
Time of recanalisation (MTE)	Date, time
mTICI score	0/1/2a/2b/3/N.A.
Imaging	CT/MRI/None
Time of CT	Date, time
Time of CT evaluation	Date, time
Time of CT angiography	Date, time
Time of CT perfusion	Date, time
Time of MRI	Date, time
Time of MRI evaluation	Date, time
CT angiography in PSC	Yes/No/N.A.
CT angiography in CSC	Yes/No/N.A.
SAH treatment	Endovascular/Surgery/None/N.A.
NIHSS value on admission	0–42
NIHSS value 24 h post-admission	0–42
NIHSS value 7 days post-admission	0–42
NIHSS value on discharge	0–42
mRS value on admission	0–6
mRS value after 7 days	0–6
mRS value on discharge	0–6
Medications given in inpatient settings	ATC subgroup
Antithrombotic therapy given in inpatient settings	Yes/No
Time of antithrombotic therapy initiation	Date, time
Rehabilitation in inpatient settings	Yes/No
Time of rehabilitation initiation	Date, time
Swallowing assessment in inpatient settings	Yes/No
Time of swallowing assessment	Date, time
Referral to rehabilitation after discharge	Inpatient level 2/Inpatient level 3/Outpatient/None
Time of inpatient discharge	Date, time
Cause of discharge	Inpatient care not required/Transfer to inpatient rehabilitation/Transfer to palliative care/Transfer to psychiatric hospital/Transfer to another hospital/Death
Death in inpatient settings	Yes/No
Death caused by stroke	Yes/No
Anticoagulants prescribed on discharge	Yes/No
Follow-up information	
Active prescription of antithrombotic medication in 1 year	ATC subgroup/N.A.
Active prescription of antithrombotic medication in 2 years	ATC subgroup/N.A.
Filled patient satisfaction survey	Yes/No

N.A., Not Applicable; ATC, The Anatomical Therapeutic Chemical Classification System; ICD-10, The International Statistical Classification of Diseases and Related Health Problems, Tenth Revision, Australian Modification; mRS, Modified Rankin Scale; TIA, Transient Ischemic Attack; PSC, Primary Stroke Centre; CSC, Comprehensive Stroke Centre; EMS, Emergency Medical Services; ER, Emergency Room; IVT, Intravenous Thrombolysis; EVT, Endovascular Therapy; CT, Computed Tomography; MRI, Magnetic Resonance Imaging; MTE, Mechanical Thrombectomy; mTICI, Modified Treatment in Cerebral Infarction scale; NIHSS, National Institutes of Health Stroke Scale.

The four HISs will undergo extensive review, and new manually entered or automatically populated fields will be added to enable automatic extraction of required variables (Table 2). This enhancement will allow for KPI monitoring directly from the primary data source, eliminating the need for manual labour and reducing human-induced bias.

## Longitudinal outcome assessment

All patients included in the Lithuanian Stroke Database will be assessed for long-term outcomes using data from information systems, linked by the unique national identification number assigned to each citizen. The SDA will carry out the linkage, pseudonymise or anonymise the data (depending on the needs of each analysis) and provide anonymised data analysis capabilities through an interactive dashboard. Additional data from other state databases can be linked on-demand through the national identification number. All of this data, at a pseudonymised or anonymised level, will be available from the SDA for research, innovation and other purposes in accordance with the national Law for Health Data Reuse and the upcoming European Union Regulation on European Health Data Space (22).

Although Lithuania does not currently utilise real-time daily updates like some other countries (23), the Lithuanian approach leverages the broad data collection capabilities of national data platforms, which gather healthcare information at multiple stages of the patient journey. These systems capture data on hospital admissions, discharge dates, diagnoses, mortality and follow up metrics such as the modified Rankin Scale (mRS) for functional outcomes and the National Institutes of Health Stroke Scale (NIHSS) for neurological assessment at discharge, providing a comprehensive picture of patient outcomes across different healthcare settings.

## Prospects

The US National Academy of Medicine proposes the implementation of Learning Healthcare Systems (LHS) – a value-based comprehensive model of care delivery for healthcare organisations to become more systematic and base knowledge on data to transition to high-value care (24). Specifically, internal data collection is proposed to be integrated with external evidence (25), hence, systematic data collection is crucial for improved care management. However, the application of this principle in stroke seems to be sparse (26). Although high-income countries such as Canada, the United States, Japan, Denmark, and Czechia have established integrated stroke systems that greatly improve stroke care quality, these systems are rare, especially in low-income countries (26).

The Lithuanian Stroke Database represents a significant step toward improving the quality of stroke care nationwide and building a national stroke LHS. With data integration, patient organisation involvement, and the implementation of feedback mechanisms, the database could inform updates to current guidelines based on real-time insights, embodying the principles of an LHS. The database will provide valuable insights into acute and long-term stroke outcomes by systematically collecting and analysing data from stroke centres nationwide. This data-driven approach allows healthcare providers to identify gaps in care, implement evidence-based interventions, and track the effectiveness of treatments over time. With the regular audit and feedback mechanisms built into the system, the database is expected to foster a culture of continuous improvement in stroke care.

In the future, collecting new data and integrating more real-time variables alongside already existing national databases will enable even faster response times for acute stroke treatment, optimising processes such as door-to-needle and door-to-reperfusion times. As the database matures, it will serve as a platform for benchmarking stroke care quality across different regions and institutions, ultimately standardising care practices in line with international guidelines. Moreover, the database can support national health policy decisions by providing comprehensive, long-term data on patient outcomes, as well as helping shape strategies for stroke prevention, rehabilitation, and secondary care.

## Administration and funding

The Lithuanian Stroke Database is part of a broader cluster project financed with the funds of the European Union’s Recovery and Resilience Facility (“Platform for data exchange and monitoring of medical clusters,” project number 09-039-P-0001) under the plan “New Generation Lithuania.” The project is managed under the national medical cluster initiative, which aims to enhance the coordination and quality of stroke care across multiple healthcare institutions. The funding supports the development of the database’s infrastructure, data management systems, and ongoing auditing processes, ensuring the project’s sustainability and long-term impact on stroke care in Lithuania. As the data are extracted automatically from structured admission and discharge documentation, no additional input or training is required from clinical staff or administrative personnel.

## Conclusion

The Lithuanian Stroke Database is a project aimed at improving stroke care nationwide. The key feature of this project is the selection of 53 KPIs through a multi-stage Delphi process involving national stroke experts who analysed international standards and guidelines. These KPIs provide a comprehensive framework for monitoring both clinical processes, such as timeliness and early rehabilitation, and outcomes, including functional recovery, mortality, and patient feedback. By implementing an automated data collection system from all Lithuanian stroke centres, the database streamlines selected performance indicator monitoring, reduces reliance on manual data entry, and minimises human-induced bias. The collected data will be available for research, innovation, and other secondary-use projects, ensuring continuous quality improvement in stroke care.

## Data Availability

The original contributions presented in the study are included in the article/Supplementary material, further inquiries can be directed to the corresponding author.
