# Supplementary material for: The Lithuanian Stroke Database: selection of national stroke care performance measures
Source: Front Neurol. 2025 May 23;16:1550539. doi: 10.3389/fneur.2025.1550539 (PMC12141015; doi:10.3389/fneur.2025.1550539)
Supplement: Supplementary file 1 [file Table_1.docx]

Supplementary Material

**Supplementary Table 1.** Lithuanian Stroke Database Steering Committee and other stakeholders, involved in the initial key performance indicator selection.

| Stakeholder | Institution | Position |
| --- | --- | --- |
| Daiva Rastenytė | The Hospital of Lithuanian University of Health Sciences Kauno Klinikos | Head of Neurology Department,  Chair of SICMC |
| Vaidas Matijošaitis | The Hospital of Lithuanian University of Health Sciences Kauno Klinikos | Head of the in-patient Neurology Unit  Stroke Section |
| Rytis Masiliūnas | Vilnius University Hospital Santaros Klinikos | Head of the Subdepartment of Emergency Neurology |
| Dalius Jatužis | Vilnius University Hospital Santaros Klinikos | Senior Neurologist at the Centre of Neurology Neurophysiology Clinic |
| Andrius Klimašauskas | Vilnius University Hospital Santaros Klinikos | Head of the Emergency Medicine Centre |
| Saulius Taroza | Klaipėda University Hospital | Head of the Neurology Centre |
| Sandra Baužaitė-Babušienė | Republican Panevėžys Hospital | Head of the Neurology I Department |
| Miglė Noreikaitė | Ministry of Health of the Republic of Lithuania | Advisor for the Specialised Healthcare Unit at the Personal Health Department |
| Greta Makauskaitė | Ministry of Health of the Republic of Lithuania | Advisor for the Health System Information Resources Development Department |
| Viktorija Buzytė | Ministry of Health of the Republic of Lithuania | Advisor for the Primary Health Care, Dentistry, and Medical Rehabilitation Department |
| Jolanta Valentienė | State Enterprise Centre of Registers | E-health Product Manager |
| Saulė Greičienė | State Data Agency | Health Data Analyst |
| Rita Gaidelytė | Institute of Hygiene | Head of the Health Statistics Unit |

SICMC **–** Stroke Integrated Care Management Committee.

**Supplementary Table 2.** Stroke Integrated Care Management Committee (SICMC) members.

| Member | Institution | Position |
| --- | --- | --- |
| Daiva Rastenytė | The Hospital of Lithuanian University of Health Sciences Kauno Klinikos | Head of Neurology Department,  Chair of SICMC |
| Dalius Jatužis | Vilnius University Hospital Santaros Klinikos | Senior Neurologist at the Centre of Neurology Neurophysiology Clinic |
| Veslava Bobrovskaja | The State Accreditation Service for Health Care Activities Under the Ministry of Health | Senior Specialist for Patient Rights and Service Quality |
| Inga Cechanovičienė | Ministry of Health of the Republic of Lithuania | Head of the Specialised Healthcare Unit at the Personal Health Department |
| Rita Gaidelytė | Institute of Hygiene | Head of the Health Statistics Unit |
| Giedrė Girdvainytė | Regional Telšiai Hospital | Temporary Assistant Director |
| Irma Jasionienė | Marijampolė Hospital | Head of the Neurology Department |
| Ilona Kajokaitė | Kaunas City Emergency Medical Services | Assistant Director for Medicine |
| Linas Masiliūnas | Republican Panevėžys Hospital | Head of the Neurology II Department |
| Rytis Masiliūnas | Vilnius University Hospital Santaros Klinikos | Head of the Subdepartment of Emergency Neurology |
| Danas Masiulionis | Tauragė Hospital | Assistant Director for Medicine |
| Miglė Noreikaitė | Ministry of Health of the Republic of Lithuania | Advisor for the Specialised Healthcare Unit at the Personal Health Department |
| Edita Pacevičienė | Republican Šiauliai Hospital | Neurologist |
| Rosida Poškienė | Alytus Region S. Kudirka Hospital | Head of the Neurology Department |
| Rita Stasevičienė | Utena Hospital | Neurologist |
| Saulius Taroza | Klaipėda University Hospital | Head of the Neurology Centre |
| Vilma Uždavinienė | Territorial Health Insurance Funds Under the Ministry of Health | Head of the Service Monitoring Unit |
| Antanas Vaitkus | The Hospital of Lithuanian University of Health Sciences Kauno Klinikos | Head of the in-patient Neurology Unit |
| Augenijus Vilimas | Republican Vilnius University Hospital | Head of the Neurology I Department for Vascular Disorders |
| Aleksandras Vilionskis | Lithuanian Stroke Association | President of the Lithuanian Stroke Association |
